# *In vitro* Infection of Primary Human Monocytes with HIV-1

**DOI:** 10.21769/BioProtoc.3289

**Published:** 2019-07-05

**Authors:** Patrick O’Connell, Yong-Hui Zheng, Andrea Amalfitano, Yasser A. Aldhamen

**Affiliations:** 1 Department of Microbiology and Molecular Genetics, College of Osteopathic Medicine, Michigan State University, East Lansing, USA; 2 Department of Pediatrics, College of Osteopathic Medicine, Michigan State University, East Lansing, USA

**Keywords:** Monocytes, HIV, Infection, SLAMF7, Immunomodulation, Neuroinflammation, FACS, Flow cytometry

## Abstract

**[Abstract**] Monocyte infection by HIV-1 is an important component of chronic HIV pathogenesis. Following infection by HIV-1, monocytes are able to cross the blood brain barrier and set up a viral reservoir in the central nervous system. Additionally, in the setting of chronic HIV-1 infection, monocytes can become activated either directly through HIV-1 infection or indirectly via HIV-1-mediated systemic immune activation. Currently, there are few studies looking at HIV-1 infection of primary human monocytes *in vitro*. Furthermore, detection of successful HIV-1 infection of monocytes can be laborious requiring an ELISA for p24 or assessing levels of HIV-1 mRNA or DNA. This protocol utilizes an HIV-1 strain expressing GFP to allow for easy quantification of HIV-1 infection by fluorescence-assisted cell sorting (FACS). By determining HIV-1 infection by FACS one can take advantage of its multiparametric nature allowing for the use of less cells and the ability to assess the expression of other markers on HIV-1^+^ and HIV-1^-^ cells in the same experiment. Additionally, this protocol could be modified to study HIV-1 infection of other cells including CD4^+^ T cells.

## Background

[**Background**] This protocol will be of use to those looking to perform studies assessing the role that myeloid cells play in HIV-1 infection. Myeloid cells, in particular, monocytes, have been recognized as crucial mediators of chronic and acute inflammation in HIV-1 infection (Burdo *et al.*, 2013; Anzinger *et al.*, 2014; Merino *et al.*, 2017). Gaining a deeper understanding of the interactions between the HIV-1 virus and monocytes will be fundamental in advancing our knowledge on how HIV-1 infection produces a chronic state of immune activation, even in the presence of combined anti-retroviral therapy (Anzinger *et al.*, 2014). With persons infected by HIV-1 now exhibiting lower death rates, we are just now seeing the long-term effects of HIV-1-induced chronic immune activation which can present as: HIV-associated neurocognitive disorder (HAND), hepatic steatosis, renal failure, hepatitis, atherosclerosis, insulin resistance, osteoporosis, and more (Cardoso *et al.*, 2013; Katlama *et al.*, 2013).

 The method presented here will be useful to both study how HIV-1 infection of monocytes alters cellular function, and to facilitate the development of novel methods to modulate or prevent HIV-1 infection of monocytes. In particular, this protocol is advantageous in that it allows for quick and easy analysis of monocyte HIV-1 infection using FACS without the need to intracellular stain for p24.

## Materials and Reagents

1. LS columns (Miltenyi Biotec, catalog number: 130-042-401)
2. Glass Pasteur pipettes, autoclaved (VWR, catalog number: 14672-200)
3. Aspirating bulb for use with Pasteur pipet (CELLTREAT Scientific Products, catalog number: 229279)
4. 50 ml conical tubes (Corning, catalog number: 430290)
5. Serological pipettes (Costar)
6. 1 ml micropipette
7. 96-well high-binding plates (Corning, catalog number: 3361)
8. Pipette tips
9. pNL-BaL-GFP recombinant HIV-1 virus
Construction of pNL-BaL-GFP HIV-1 vector. The pNL4-3env(-)GFP, which encodes full-length NL4-3 HIV-1 proviral DNA with a frameshift in env and expresses GFP in place of nef, was described previously (He *et al.*, 1997). The CCR5-tropic HIV-1 proviral clone, R9BaL, was generated from R9 by replacing an EcoRI-XhoI fragment from the CCR5-tropic HIV-1 strain, BaL, which contains the entire *env* coding region (Gallay *et al.*, 1995). pNL-BaL-GFP was constructed by replacing a SalI-BamHI fragment in pNL4-3env(-)GFP with that from R9BaL.
10. Antibodies:
  - CD14, PE-Cy7 (clone: 61D3) (Thermo Fisher, catalog number: 25-0149-42)
  - CD16, BV510 (Clone: 3G8) (BD BioSciences, catalog number: 563830)
  - SLAMF7, PE (Clone: 162) (BioLegend, catalog number: 331806)
  - CCR5, unconjugated (Clone: 2D7) (BD BioSciences, catalog number: 555990)
11. Viability dye: Live/Dead Violet (Thermo Fisher, catalog number: L34964)
12. CD14 microbeads, human (Miltenyi Biotec, catalog number: 130-050-201)
13. Fresh human buffy coat (Gulf Coast Regional Blood Center, Texas, USA)
14. Lympholyte-H (Cedarlane Labs, catalog number: CL5020)
15. DPBS without calcium or magnesium (Gibco, catalog number: 14190250)
16. RPMI 1640 (Gibco, catalog number: 11875093)
17. ACK Lysis buffer (Gibco, catalog number: A1049201)
18. Fetal Bovine Serum (FBS) (Atlanta Biologicals, catalog number: S11550)
19. Antibiotic/Antimycotic (Penicillin, Streptomycin, Fungizone) (PSF) (Gibco, catalog number: 15240062)
20. Ethylenediaminetetraacetic acid (EDTA) (Millipore Sigma, catalog number: E6758)
21. BSA (Millipore Sigma, catalog number: A9056)
22. Sodium azide (Millipore Sigma, catalog number: S2002)
23. Fixation buffer from BD Fix/Perm kit (BD Biosciences, catalog number: 554714)
24. Cross-linking antibody of choice (for stimulation of monocytes during HIV-1 infection)
25. FACS solution (see Recipes)
26. MACS solution (see Recipes)
27. Complete RPMI solution (see Recipes)

## Equipment

1. Biosafety cabinet
2. Gun pipette
3. QuadroMACS magnetic cell separator (Miltenyi Biotec, catalog number: 130-090-976)
4. LSRII flow cytometer (BD Biosciences)
5. Tabletop centrifuge with swinging bucket rotor
6. Automated cell counter (Countess) (Invitrogen)

## Software

1. FlowJo version 10.5 (https://www.flowjo.com)
2. GraphPad Prism V7 (https://www.graphpad.com/scientific-software/prism/)

## Procedure

1. Isolate PBMCs from a fresh human buffy coat using Lympholyte-H under sterile conditions.
*Note: Perform all the following steps in a sterile biosafety cabinet.*
  - Allow Lympholyte-H to equilibrate to room temperature. This is important to ensure efficient isolation of peripheral blood mononuclear cells (PBMCs).
  - Split approximately 50 ml buffy coat evenly into four 50 ml conical tubes (12.5 ml per tube).
  - Add 22.5 ml DPBS to each tube containing pure buffy coat to bring the volume up to 35 ml. (The ratio of DPBS to buffy coat should be greater than 1:1.)
  - Shake vigorously (or vortex) the 50 ml conical tubes for 10 s. This is important to prevent clumping of cells which will affect isolation and can cause RBC contamination.
  - Shake lympholyte-H vigorously just before use.
  - Work in a biosafety cabinet with lights off.
*Note: Lympholyte-H is light sensitive.*
  - Place a sterile glass Pasteur pipette into the bottom of a 50 ml conical tube containing the blood/DPBS mixture. Add Lympholyte-H to the glass Pasture pipette using a gun pipette. This is best accomplished using a 10 ml stripette with the power setting on the gun pipette set to “gravity”. A total of 10 ml of Lympholyte-H should be added per 50 ml conical tube.
  - Lympholyte-H should immediately begin to form a layer on the bottom of the conical tube, clearly separated from the buffy coat (Figure 1.h).
  - Add a total of 10 ml lympholyte-H to each 50 ml conical tube.
  - Repeat for each 50 ml conical tube. The final volume in each tube will now be 45 ml.
  - Spin 50 ml conical tubes at 1,800 *× g*, 24 °C, no break, maximum acceleration, and minimum deceleration, for 30 min in any swinging bucket benchtop centrifuge.
  - Attach a rubber suction bulb to a fresh Pasteur pipette. Carefully remove the middle lymphocyte band (approximately 5 ml) from each 50 ml conical tube. This band will be cloudy, may contain some RBCs, and will be located above the pelleted RBCs (at the bottom of the tube) (Figure 1.I).
  - Transfer these cells to a fresh 50 ml conical tube.
  - Repeat this for each of the four 50 ml conical tubes.
  - Add DPBS to a final volume of 50 ml into each new 50 ml conical tube containing pure lymphocytes.
  - Spin the tube at 1,500 *× g*, 24 °C, full break for 5 min (“full break” means the centerfuge break is on).
  - Discard the supernatant.
  - Resuspend each pellet in 2 ml ACK Lysis buffer using a 1 ml micropipette. Ensure pellet is fully resuspended.
  - Incubate cells in ACK lysis buffer for exactly 6 min at room temperature.
  - Top with DPBS, spin the tube at 900 *× g*, 24 °C, full break for 5 min.
  - Discard the supernatant.
  - Resuspend in complete RPMI pre-warmed to 37 °C using a 1 ml micropipette. Ensure cells are in a single cell suspension. Add a total of 10 ml of media per buffy coat. At this stage, you can combine cells from the 4 separate fractions (Figure 1.v).
  - Prepare 1:100 dilution of cells into complete RPMI and count on a Countess (or your preferred method) per manufacturer’s instructions.
  **Figure 1.**
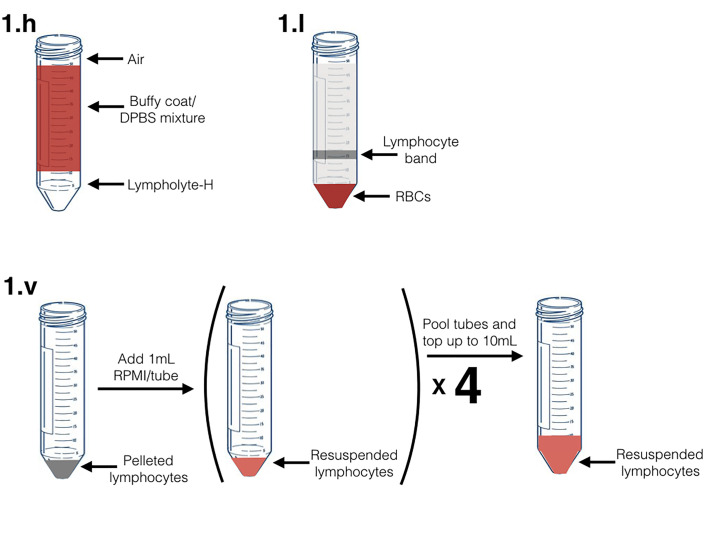
Graphical diagram for selected steps in part 1 of procedure (PBMC) isolation. Figure labels correspond to indicated steps in PBMC isolation procedure.
2. Isolate CD14^+^ monocytes by positive selection (CD14 microbeads, Miltenyi Biotec) per manufacturer recommendation using MACS buffer.
*Note: Incubate cells with anti-CD14 microbeads for 25 min to increase the purity of isolated monocytes.*
3. Check the purity of monocytes before proceeding. Stain cells (pre and post-isolation) with CD14 PE-Cy7 mAb and Live/Dead Violet viability dye per manufacturer’s instructions and as previously described (O'Connell *et al.*, 2018). Run cells on LSRII flow cytometer (or other flow cytometers) and determine % of cells CD14^+^ that are viable (Figure 2). If isolated monocyte samples are less then 90% CD14^+^ it is recommended not to proceed. Should monocyte purity be < 90% you may return the monocytes to MACS buffer (if you have already resuspended them in complete RPMI) and pass them through a fresh MACS LS column exactly as performed in Step 2.
  **Figure 2.**
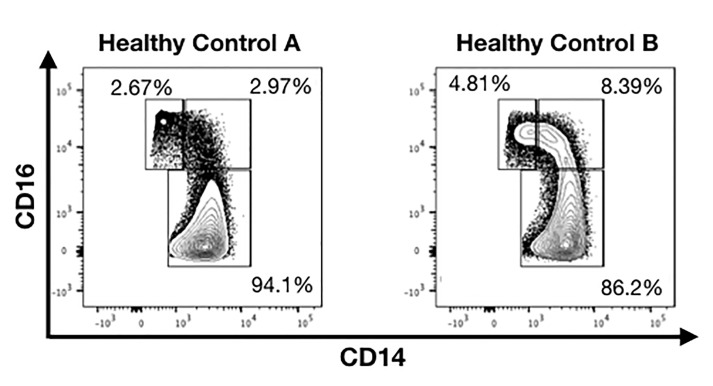
FACS plots showing subtypes of monocytes between 2 healthy blood donors . Healthy control B has considerably more CD16^+^ monocytes. Cells shown are gated on singlets, myeloid cell population (based on FSC and SSC), living, and expressing CD14.
4. Resuspend monocytes in complete RPMI and plate in 96-well high-binding cell culture plates at 2 x 10^5^ cells/well.
5. If activation of a monocyte cell surface receptor is desired (*e.g*., SLAMF7 [O'Connell *et al.*, 2018]), coat high-binding plate with an antibody against the desired receptor for 24 h at 4 °C beforehand. Dilute cross-linking antibody in DPBS before adding it to wells for cross-linking. Wash wells 3 times with DPBS before addition of cells and make sure wells do not dry out at any point.
6. As a positive control, if using an R5-tropic HIV-1 virus, add 10 μg/ml of the CCR5-blocking mAb (2D7) per appropriate well. Wells containing this soluble mAb should retain appropriate concentrations of this mAb at all media changes.
7. After 24 h of cell culture at 37 °C and 4% CO_2_, remove media and add fresh media containing 190 pg/ml pNL-BaL-GFP.
8. After 4 h, wash pNL-BaL-GFP infected cells 2 times with complete RPMI.
9. Resuspend cells in 200 μl complete RPMI/well and culture at 37 °C and 4% CO_2_.
10. Every 24-36 h remove an aliquot of cells (*i.e.*, one well) per condition and wash them with FACS.
11. Stain cells with CD14, CD16, Live/Dead Violet viability dye, and any other antibodies (*i.e.*, SLAMF7) desired as previously described (O'Connell *et al.*, 2018).
12. Fix cells for 20 min at room temperature, protected from light, by adding 200 μl of BD Fixation buffer per well and thoroughly resuspending cells.
13. Wash cells twice with FACS.
14. Analyze stained cells on an LSRII (or similar flow cytometer) to determine (%) CD14^+^ Live/Dead Violet- GFP^+^ cells.
*Note: Cells should be analyzed within 24 h of picking them up to ensure GFP signal is not lost. Fixation will not affect GFP signal over short term (less than 24 h), but prolonged fixation will degrade GFP.*
15. GFP^+^ cells are considered to be HIV-1^+^ as all infected cells using this virus will express GFP. The addition of CD14 and viability markers ensure that cells analyzed are monocytes (*i.e.*, CD14^+^) and living (*i.e.*, Live/Dead Violet-).

## Data analysis

1. Analyze all data using FlowJo v10.5 or greater (or other FACS analysis software per your preference).
2. Analyses should be conducted by assessing the (%) CD14^+^ Live/Dead Violet- GFP^+^ cells per condition. Inclusion of other markers can be included to allow for additional analyses.
3. Example analyses are presented in Figure 3.
4. When comparing infectivity between groups, use a student’s *t*-test or one-way ANOVA as appropriate.
  **Figure 3.**
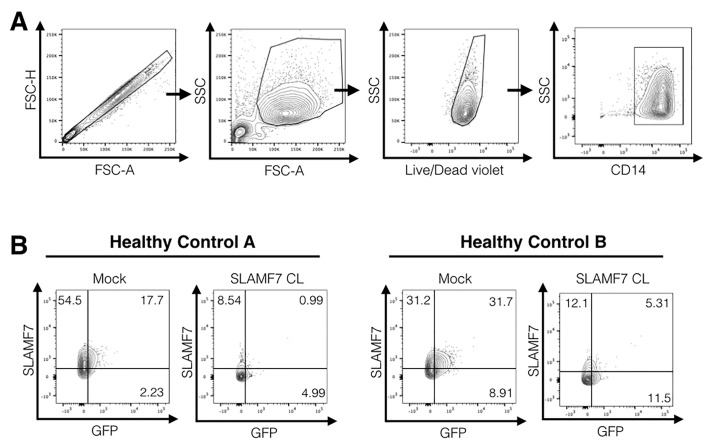
Example FACS analysis. A. Representative gating scheme used for analysis of monocyte infectivity. B. Representative FACS plots showing examples of positive and negative GFP monocytes from the CD14^+^ gate above. Mock conditions are monocytes infected with pNL-BaL-GFP and no immune cell receptor cross-linking. Note how HIV-1^+^ monocytes tend to be positive for the SLAMF7 receptor and activation of the SLAMF7 receptor via cross-linking prevents monocyte infection. Co-expression analyses like this can be performed with any other marker of interest. CL; cross-linking.
5. Data can be displayed as an X-Y graph with connecting lines (O'Connell *et al.*, 2018, Figure 6d) and also as a FACS dot plot to provide additional support for your results (O'Connell *et al.*, 2018, Figure 6d).

## Notes

1. This protocol has been reproducible in our hands, and we have only noticed variability in the degree of infectivity between individual blood donors.
2. Refresh media on cultured monocytes every 48 h.
3. Culture cells for a maximum of 7 days. Primary human monocytes will not typically survive more than 7 days post-isolation.
4. Monocytes should show a time-dependent increase in the number of HIV-1^+ ^cells until approximately 6-7 days, at which point the infectivity should level out as monocytes reach their terminal ex-vivo lifespan.
5. The efficiency of HIV-1 infection of monocytes will vary between subjects and is dependent on the percent of CD16^+^ monocytes. Individuals with higher levels of CD16^+^ monocytes will show a greater percent of HIV-1^+^ monocytes (Figures 2 and 3) (Tuttle *et al.*, 1998; Ellery *et al.*, 2007).

## Recipes

1. FACS solution
  500 ml DPBS (1 bottle)
  1% (W/V) BSA
  0.1% (W/V) sodium azide
2. MACS solution
  500 ml DPBS (1 bottle)
  0.5% BSA
  2 mM EDTA (used for CD14 isolation)
3. Complete RPMI solution
  500 ml RPMI 1640
  10% FBS
  1% PSF
